# A differential transcriptional profile by *Culex quinquefasciatus* larvae resistant to *Lysinibacillus sphaericus* IAB59 highlights genes and pathways associated with the resistance phenotype

**DOI:** 10.1186/s13071-019-3661-y

**Published:** 2019-08-20

**Authors:** Tatiana Maria Teodoro Rezende, Antonio Mauro Rezende, Gabriel Luz Wallau, Crhisllane Rafaele Santos Vasconcelos, Osvaldo Pompílio de-Melo-Neto, Maria Helena Neves Lobo Silva-Filha, Tatiany Patrícia Romão

**Affiliations:** Instituto Aggeu Magalhães-FIOCRUZ, Av. Moraes Rego s/n Cidade Universitária, Recife, PE 50740-465 Brazil

**Keywords:** Biolarvicides, Binary toxin, Cry48Aa/Cry49Aa, Receptors, Cqm1, Transcriptome

## Abstract

**Background:**

The study of the mechanisms by which larvae of the *Culex quinquefasciatus* mosquito survive exposure to the entomopathogen *Lysinibacillus sphaericus* has benefited substantially from the generation of laboratory-selected colonies resistant to this bacterium. One such colony, RIAB59, was selected after regular long-term exposure of larvae to the *L. sphaericus* IAB59 strain. This strain is characterized by its ability to produce the well known Binary (Bin) toxin, and the recently characterized Cry48Aa/Cry49Aa toxin, able to kill Bin-resistant larvae. Resistance to Bin is associated with the depletion of its receptor, Cqm1 α-glucosidase, from the larvae midgut. This study aimed to identify novel molecules and pathways associated with survival of the RIAB59 larvae and the resistance phenotype.

**Methods:**

A transcriptomic approach and bioinformatic tools were used to compare the profiles derived from the midguts of larvae resistant and susceptible to *L. sphaericus* IAB59.

**Results:**

The RNA-seq profiles identified 1355 differentially expressed genes (DEGs), with 673 down- and 682 upregulated transcripts. One of the most downregulated DEGs was *cqm1*, which validates the approach. Other strongly downregulated mRNAs encode the enzyme pantetheinase, apolipoprotein D, lipases, heat-shock proteins and a number of lesser known and hypothetical polypeptides. Among the upregulated DEGs, the top most encodes a peroxisomal enzyme involved in lipid metabolism, while others encode enzymes associated with juvenile hormone synthesis, ion channels, DNA binding proteins and defense polypeptides. Further analyses confirmed a strong downregulation of several enzymes involved in lipid catabolism while the assignment of DEGs into metabolic pathways highlighted the upregulation of those related to DNA synthesis and maintenance, confirmed by their clustering into related protein networks. Several other pathways were also identified with mixed profiles of down- and upregulated transcripts. Quantitative RT-PCR confirmed the changes in levels seen for selected mRNAs.

**Conclusions:**

Our transcriptome-wide dataset revealed that the RIAB59 colony, found to be substantially more resistant to Bin than to the Cry48Aa/Cry49Aa toxin, developed a differential expression profile as well as metabolic features co-selected during the long-term adaptation to IAB59 and that are most likely linked to Bin resistance.
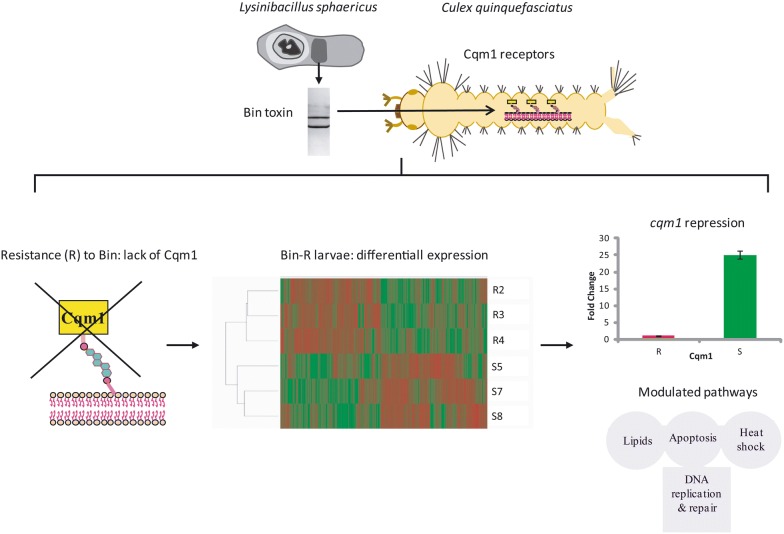

**Electronic supplementary material:**

The online version of this article (10.1186/s13071-019-3661-y) contains supplementary material, which is available to authorized users.

## Background

*Culex quinquefasciatus* is one of the most abundant mosquito species associated with urban areas, particularly those which are characterized by precarious sanitation. This antropophilic species is a known vector of human pathogens and it has been the target of control programmes worldwide. Biolarvicides based on the entomopathogenic bacterium *Lysinibacillus sphaericus* are some of the most effective agents used to control *Cx. quinquefasciatus* populations [1]. Binary (Bin) toxin, its major insecticidal factor, is highly active against larvae from this mosquito species and displays long persistence in its breeding sites [2]. However, as most insecticides that have been used in the field, its effectiveness faces the emergence of resistant mosquito populations [3]. Therefore, the major challenge for *L. sphaericus* utilization is to overcome the resistance that can be selected against the Bin toxin, the active ingredient of the commercially available biolarvicides.

Bin is synthesized as a heterodimeric protoxin and its two subunits, BinA (~43 kDa) and BinB (~51 kDa), act in synergy to reach optimal toxicity in equimolar amounts [4]. Its larvicidal activity against *Cx. quinquefasciatus* larvae requires protoxin ingestion, midgut processing of protoxin into toxin by serine-proteases [5] and specific binding to midgut-bound receptors [6]. These receptors were identified as Cqm1 glycosylphosphatidylinositol (GPI) anchored α-glucosidases [7, 8], maltases that may play roles in the metabolism of carbohydrates [9, 10]. Binding of the Bin toxin to the Cqm1 receptors allows it to form pores and penetrate the membranes of midgut-cells [11–13]. Larval intoxication by Bin induces cell autophagy and cytoplasmatic vacuolization, one of the most prominent cytopathological alterations recorded in the midgut epithelium of treated individuals [14–16]. Mitochondrial damages have also been recorded as a marked effect of Bin toxin and these have been recently demonstrated to be involved with the activation of the intrinsic apoptosis pathway [17].

Resistance to the Bin toxin has been recorded in several occasions [18–23] and the major mechanism responsible for that is the failure of this toxin to bind to the midgut-cells, mostly due to the lack of Cqm1 receptors [19, 20, 24]. Further studies defined that mutations in the *cqm1* gene can disrupt the expression of full-length GPI anchored proteins and cause high resistance ratios (> 5000-fold) [7, 25–29]. Laboratory-selected colonies highly resistant to the Bin toxin have been used as models to investigate resistance and to screen for other bacterial strains and toxins that could be active against these larvae and overcome Bin resistance [21, 23]. In this context, the *L. sphaericus* IAB59 strain was thus found to be toxic to Bin-resistant larvae [21, 30] and able to produce the Bin toxin as well as Cry48Aa/Cry49Aa, another binary toxin responsible for the larvicidal effect against those larvae [31, 32]. Previous studies have indicated that the Cry48Aa/Cry49Aa toxin was able to interact with receptors from the midgut of susceptible [33] and also with Bin resistant larvae lacking the Cqm1 receptor [34]. Specific receptors involved in this interaction with Cry48Aa/Cry49Aa are being studied and potential candidates have been recently tentatively identified [35]. The *L. sphaericus* IAB59 strain was also employed to select a laboratory resistant *Cx. quinquefasciatus* colony (RIAB59) and, after 72 generations, a high resistance level was achieved to the IAB59 crystals-spores used to treat the larvae throughout the selection process [21, 36]. During the selection procedure, a high resistance specific to the Bin toxin arose much earlier [21, 37] than the resistance to the whole set of IAB59 toxins [36]. In fact, the resistance level of the RIAB59 larvae to Cry48Aa/Cry49Aa only was much lower than that recorded for the Bin toxin [34].

The high level of resistance of the *Cx. quinquefasciatus* RIAB59 larvae to the Bin toxin [36] was previously characterized and shown to be associated with the *cqm1*_*REC*_ allele. A frame shift change within this allele leads to the loss of the GPI-anchored Bin receptors in the midgut epithelium [36, 38]. Individuals from the RIAB59 colony are homozygous for the *cqm1*_*REC*_ and the resistance is recessively inherited [36]. The disruption of Cqm1 expression also represents the lack of a maltase that can presumably lead to an impact on the digestion of carbohydrates by the insect larvae. Nevertheless this colony has been successfully maintained in the laboratory under intermittent selection with *L. sphaericus* IAB59 strain for more than 200 generations. Assessment of biological parameters as fecundity, fertility and pupae weight in RIAB59 individuals did not reveal significant differences in comparison to a susceptible reference colony [36, 38]. These results suggest that those individuals developed mechanisms to balance the potential costs associated to the resistance phenotype. In this context we employed here a differential expression analysis at the transcriptome scale comparing the RIAB59 larvae with individuals from a susceptible colony.

## Methods

### *Culex quinquefasciatus* colonies

CqSLab is a laboratory reference colony susceptible to insecticidal compounds while RIAB59 is a resistant laboratory colony selected after continuous exposure to the *L. sphaericus* IAB59 strain as described in previous studies [36, 38]. The IAB59 strain produces Binary 1 toxin, named Bin1 [39], as well as Cry48Aa/Cry49Aa toxins [31]. Bin1 has a similar larvicidal activity and capacity to bind to midgut receptors as Binary 2, produced by other *L. sphaericus* strains, such as 2362, 1593 and C3–41 [37]. The strong ratio of resistance (RR) to the Bin toxin (RR > 5000) achieved by the RIAB59 colony is known to be due to the individuals being homozygous to the *cqm1*_*REC*_ allele [38], therefore not being able to express the midgut bound Cqm1 receptor [7]. Since the resistance to *L. sphaericus* IAB59 has been achieved [36] this colony has been exposed to the bacterium every five generations, to periodically verify the status of *in vivo* resistance. In this study larvae from generation F_194_ were used, with the last exposure to *L. sphaericus* IAB59 having taken place in generation F_189_. The larvae samples collected for this analysis from the two colonies were thus not directly exposed to the *L. sphaericus* IAB59. Both susceptible and resistant colonies have been maintained in the insectarium of the Instituto Aggeu Magalhães (IAM)-FIOCRUZ for more than five years under controlled conditions of 26 ± 1 °C, 70 % relative humidity and a 14h:10h (light:dark) photoperiod. Larvae were reared in dechlorinated water and fed with cat food (Friskies®, Nestlé Purina,  Ribeirão Preto, Brazil). Adults were maintained on a 10% sugar solution and females were also artificially fed with defibrinated rabbit blood.

### RNA extraction, mRNA library construction and sequencing

Total RNA from three pools of 20 larvae midguts, from each colony, was extracted using an RNeasy Mini Kit (Qiagen, Hilden, Germany), following the manufacturer’s instructions. RNA purity and concentration were assessed using a NanoDrop 2000™ spectrophotometer (Thermo Fisher Scientific, Waltham, USA) and Qubit 2.0 Fluorometer (Thermo Fisher Scientific). The RNA integrity was evaluated by agarose gel electrophoresis. Paired-end libraries were prepared from total RNA using the TruSeq Stranded mRNA Library Prep kit (Illumina, San Diego, USA), following standard procedures, and sequenced using a MiSeq™ Reagent Kit V3 (Illumina, San Diego, USA; 150 cycles) on an Illumina MiSeq Sequencer of the IAM-FIOCRUZ.

### RNA-seq data analysis

The quality of the sequenced reads was checked applying the FastQC tool (www.bioinformatics.babraham.ac.uk/projects/fastqc/) and we observed that, on average, all bases had Phred scores higher than 30, hence removal of low quality reads was not needed. Each library was then mapped against the genome assembly of the *Cx. quinquefasciatus* Johannesburg strain, CpipJ2 (file: culex-quinquefasciatus-johannesburgscaffoldscpipj2.fa). This was downloaded from the VectorBase database (https://www.vectorbase.org/), applying STAR aligner v.2.5.3 [40] with default parameters, except for the quantMode option which generates a GeneCounts file considering stranded libraries. The data on gene counts were next loaded to the R environment and all replicates organized in a matrix, which was transformed to log2 scale using the function rlogTransformation from the *DESeq2* package [41]. This matrix was used as input to the prcomp function in order to perform the principal components analysis (PCA). The function plot was then applied to plot the first and second principal components calculated from each sample. The R package *DESeq2* was used to perform the differential expression analysis, considering only genes represented by at least five reads for all three biological replicates, in at least one condition (resistant or susceptible). Genes with absolute values of log2 fold changes equal or greater than 1 and with FDR corrected *P*-values lower than 0.05 were selected for further analyses. It is worth noting that the real fold change value can be obtained by calculating the log2 fold change value [e.g. log2 fold change(sensitive/resistant) = 4 can be converted as 2^|4|^ = 16; this means a gene expression rate 16 times higher/lower]. The normalized read counts for each gene analyzed in all samples was next used as input to the function heatmap.2 from the *gplots* R package in order to creat a heatmap visualization from the expression data. In addition, the same data was also used to create a MA plot using the function plot, where the differentially expressed genes were highlighted. In order to further explore and refine the annotation of several DEGs tagged as hypothetical proteins in the *Cx. quinquefasciatus* genome, three additional annotation steps using Blastx alignments with default parameters were performed. mRNA nucleotide sequences from all DEGs were used as queries against the most recent versions of the following databases: (i) UniProtKB curated database; (ii) non-redundant database from NCBI retaining only the top hit for the annotation; and (iii) non-redundant database from NCBI using Blast2GO [42] retaining the top five hits for annotation. The identity of some genes was also obtained by manual annotation (Additional file 1: Table S1). All databases were downloaded up to July 12th 2018.

The *STRINGdb* R package [43] was used to assign Kyoto Encyclopedia of Genes and Genomes (KEGG) [44] pathway terms to the *Cx. quinquefasciatus* differentially expressed genes (DEGs) (http://www.vectorbase.org). The *Pathview* package [45] was then applied to map the DEGs and their fold change measures to the enriched KEGG pathways. In addition, KEGG terms were employed to perform an enrichment functional analysis of those genes using the function get_enrichment from the *STRINGdb* R package. Here, it is worth to mention the background frequency of KEGG pathways for the *Cx. quinquefasciatus* genome comes from the STRING database (https://string-db.org/). Furthermore, the DEGs were also visualized into protein networks present at STRING database. The protein networks built from the lists of DEGs were analyzed using the software Cytoscape v.3.5.1 (http://www.cytoscape.org) along with the plug-in AutoAnnotate v.1.2 (http://www.baderlab.org/Software/AutoAnotate). Here, the networks were clustered using the algorithm Community Cluster (GLay) since it does not demand a weighted network. The cluster annotations were assigned based on the three most frequent words present in the Vectorbase annotation of the protein members from each cluster.

### qRT-PCR validation of RNA-seq data

The total RNA used in this assay was the same as described above (RNA extraction, mRNA library construction and sequencing). The reactions were performed using a QuantiTect® SYBR Green RT-PCR^®^ Kit one step (Qiagen), following the manufacturer’s instructions and using specific primers for each gene selected (Additional file 2: Table S2). *18S* was used as an endogenous control gene [46]. The samples were analyzed in a QuantStudio® 5 System (Thermo Fisher Scientific) and the relative quantification was performed using Applied Biosystems™ Analysis software, Relative Quantification Analysis Module v.3.3 [47]. Means and standard errors from three biological replicates from each colony were compared using Studentʼs t-test in GraphPad Prism v.5.0.0 for Windows (GraphPad Software Inc., San Diego, USA), considering a *P*-value < 0.05 statistically significant.

## Results

### Overview of the RIAB59 resistant colony and the sequencing results

The *Cx. quinquefasciatus* RIAB59 colony selected for this study has been previously shown to display a strong and stable resistance ratio (RR) to the Bin toxin (RR > 5000-fold) [36, 38]. The specific resistance of this colony to the Cry48Aa/Cry49Aa toxin was also previously reported [34], but it was first re-evaluated here and quantified prior to the sequencing effort. The diagnostic bioassays showed that the RR between the LC_50_ of Cry48Aa/Cry49Aa for the RIAB59 larvae and that for the susceptible individuals was approximately 15-fold (data not shown). Early fourth-instar larvae from the resistant RIAB59 colony (without recent exposure to the IAB59 bacterium), as well as from a susceptible *Cx. quinquefasciatus* colony from which it was derived (CqSLab), were then harvested and had their midguts dissected and subjected to RNA extraction followed by whole transcriptome shotgun sequencing (RNA-seq). A total of 19,288,276 reads were sequenced and mapped against the *Cx. quinquefasciatus* genome. This analysis yielded 7045 genes (data not shown) that fulfilled threshold parameters of being represented by at least five reads from each of three biological replicates for either the resistant or the susceptible colonies. These correspond to roughly 36% of the total gene repertoire of the annotated *Cx. quinquefasciatus* genome. Information on the sequencing data and libraries is presented in Table 1.

**Table 1:** Statistics for the sequenced RNA-seq libraries of *Culex quinquefasciatus* larvae from a *Lysinibacillus sphaericus* RIAB59 resistant colony and a susceptible one

Sample	Resistant		Susceptible	
	No. of reads	%	No. of reads	%
Total reads	9,699,067		9,589,209	
Total base pairs	1,454,860,050		1,438,381,350	
Total mapped	9,219,975	95.06	9,171,639	95.65
Unique mapped	7,800,201	84.60	7,061,254	76.99
Multiple mapped	1,419,774	15.40	2,110,385	23.01
Unmapped	478,663	4.94	417,804	4.35
Total mapped in genes	6,402,370	82.08	5,642,580	79.91
Total mapped no gene	1,364,484	17.49	1,385,678	19.62

**Fig. 1:**
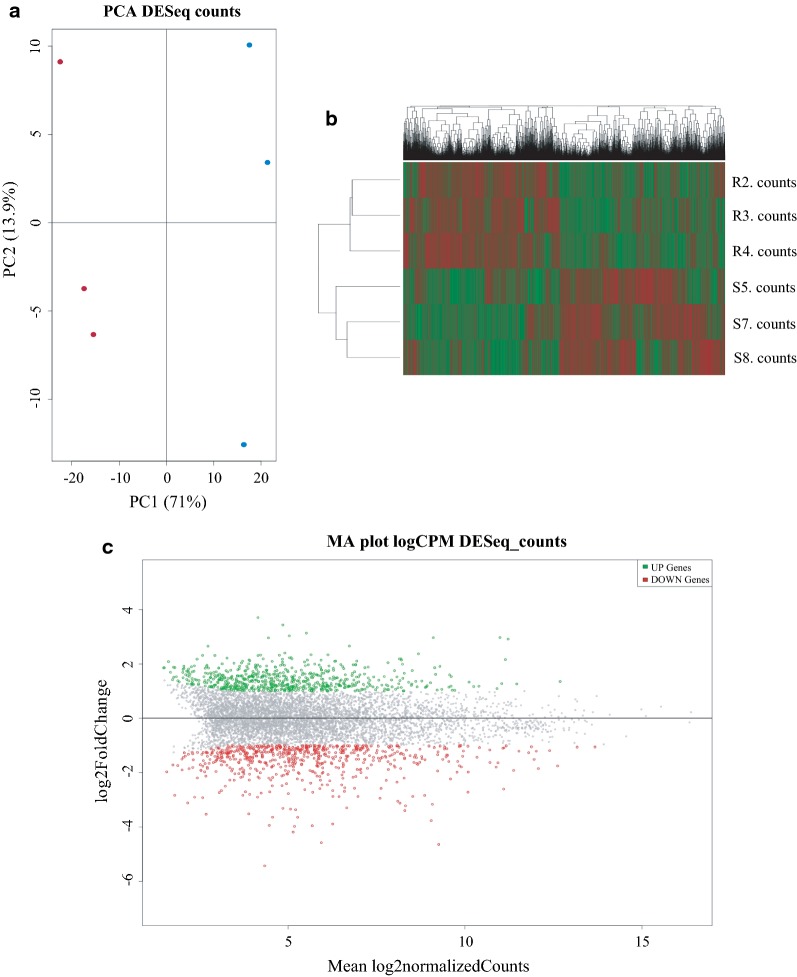
Gene expression profile generated by RNA-seq comparing the *Culex quinquefasciatus* larvae from a *Lysinibacillus sphaericus* resistant (R) and susceptible (S) colonies. **a** Principal components analysis (PCA) of the gene expression profile. **b** Heatmap for upregulated (green) and downregulated (red) genes in biological replicates from resistant (R2, R3, R4) and susceptible larvae (S5, S7, S8). **c** MA plot of differentially expressed genes. Upregulated and downregulated genes are marked in green and red, respectively

To compare the overall profile of gene expression by the larvae from the two colonies, a principal components analysis (PCA) was first carried out with the sets of annotated genes from them. Two distinct profiles of gene expression consistently reproduced with the biological replicates used were observed (Fig. 1a). Next, a heatmap was also generated comparing their overall gene expression and distinct profiles were confirmed between the two colonies which were reproduced by the biological replicates (Fig. 1b). These results are consistent with the resistance to IAB59 being associated with differential expression of a significant number of genes. It is important to notice, however, that the differential expression revealed by this study was not an induced response derived from larvae challenged with *L. sphaericus* IAB59 but a constitutive profile which is likely to result from an adaptation to the selection process.

### Landscape analysis of differentially expressed transcripts in the RIAB59 colony

To individually assess the involvement of differentially expressed genes (DEGs) in the *Cx. quinquefasciatus* resistance to the IAB59 toxins (mainly Bin), a compilation of the DEGs found comparing the resistant and susceptible colonies was then carried out. The overall distribution of the genes found after sequencing and annotation according to the log2 fold changes in number of reads observed between the resistant and susceptible colonies is represented in Fig. 1c. A total of 1355 DEGs with absolute values of log2 fold change ≥ 1 and *P-*value ≤ 0.05 were identified (Additional file 1: Table S1), with 673 of those genes downregulated in the RIAB59 colony while 682 were upregulated. Most of the DEGs identified varied within a range of two- to four-fold in the number of reads (log2 fold changes between 1 and 2), although several were found with much greater changes in expression (log2 fold changes ≥ 3). After an effort to identify the DEGs using the automatic annotation available at the VectorBase database as well as blast searches against Uniprot and GenBank databases, roughly 90% of the DEGs were identified while only 158 out of 1355 DEGs were found to encode hypothethical proteins. From the set of identified DEGs, one of the greatest scores of downregulation for the RIAB59 colony, with a log2 fold change of 4.65 (25-fold decrease) (*P* = 9.14E-304), is the *cqm1* gene (CPIJ013173; annotated as “Neutral & basic aminoacid transport protein rBAT” in VectorBase), directly responsible for the high resistance phenotype to the Bin toxin. Considering its role as the Binary toxin receptor and the fact that its absence from the larvae midgut has been previously linked to resistance to *L. sphaericus*, the gene encoding the Cqm1 α-glucosidase is a robust marker for this phenotype. Here, the identification of its mRNA within the most downregulated transcripts from the resistant colony confirms not only the resistance phenotype to Bin but also the validity of the methods applied in this study.

### Identification of the top differentially expressed genes (DEGs)

From the 1355 DEGs, those genes showing log2 fold changes greater than 3 and significant *P*-values (< 0.05) are highlighted in Tables 2 and 3. For clarity and simplification, when multiple genes were found coding for identical or nearly identical proteins, the IDs for all genes are indicated but only the values for the gene with the highest fold change are shown. Among the downregulated DEGs in the RIAB59 colony (Table 2), the gene encoding the Cqm1 α-glucosidase is found as the fourth most downregulated. To assess whether other changes in mRNA expression could help pinpoint specific proteins as potential receptors for the Cry48Aa/Cry49Aa toxin, a search for possible toxin ligands whose expression differed significantly between the susceptible and resistant colonies was first carried out. None of the candidates indicated by a previous study [35] showed differential expression except for two: the vanin 1/pantetheinase precursor, with the highest level of downregulation seen; and the apolipoprotein D transcripts (Table 2). A pantetheinase, an enzyme that acts in the catalysis of D-pantetheine into pantothenate (vitamin B5) [48], has been recently identified as one of several ligands of the Cry48Aa/Cry49Aa toxin [35], while apolipoprotein D has previously been found as a ligand for the Cry8Ea toxin in a coleopteran [49]. However, these proteins seem be rather involved in other metabolic features of the resistant larvae than being receptors, as will be further discussed. Likewise, other downregulated DEGs listed in Table 2 code for proteins that are likely not involved with specific binding by the toxin, but nevertheless may be relevant for the resistance phenotype. An example is the transcript coding for an ankyrin, a transmembrane protein belonging to pathways involved in pathogen recognition, activation of defense responses, macroautophagy and membrane transport [50–53]. Two chitin binding proteins showed substantial upregulation (CPIJ014193) and downregulation (CPIJ014195) and, although the specific role of these proteins remains unclear, they are known to participate in bacteria-host interactions during pathogen infection [54, 55]. Other strongly downregulated genes include two lipases, a fatty acid hydroxylase, heat-shock proteins, cytochrome P450 and glutathione S-transferase homologues. Some of these and several other genes found to be under strong downregulation code for proteins that are localized to the mitochondria with at least one of those involved in apoptosis, a pathway that might also be associated with yet another gene found within this list, coding for a p53 and DNA damage-regulated polypeptide. Various genes coding for hypothetical polypeptides and proteins less well known are also listed in Table 2, but their roles need further investigation.

**Table 2:** Top most downregulated genes in the *Culex quinquefasciatus* larvae from the RIAB59 resistant colony, revealed by the RNA-seq analysis

Gene_id	Description	Log2 fold change	*P*-value
CPIJ017593/CPIJ017592	Vanin 1-pantetheinase precursor	7.13	1.51E−49
CPIJ001035	Lipase member H	5.43	2.23E−45
CPIJ016846	Cytochrome P450 paralogue	5.14	1.11E−23
CPIJ013173	Cqm1-Maltase 2	4.65	9.14E−304
CPIJ011081/CPIJ011083	Heat-shock 70 B2	4.58	1.90E−13
CPIJ002679	Glutathione S-transferase theta-2	4.57	2.83E−16
CPIJ018744	Ankyrin-2,3	4.44	8.08E−16
CPIJ006512	Hypothetical protein	4.35	1.10E−13
CPIJ014435	Cuticle protein 38-like	4.33	2.85E−16
CPIJ009045	Fatty acid hydroxylase superfamily	4.19	1.67E−39
CPIJ015726/CPIJ015727	Apolipoprotein D, putative	4.10	6.54E−13
CPIJ005154	Nuclear transport factor 2	4.06	4.14E−15
CPIJ002783	AN1-type zinc finger 2B	3.97	2.52E−12
CPIJ014193	Chitin-binding protein	3.95	2.18E−51
CPIJ008858	STRK3/rubber oxygenase	3.94	6.51E−29
CPIJ004068	UBASH3A homolog	3.88	8.78E−13
CPIJ011433	Chymotrypsin BI	3.77	2.73E−35
CPIJ017634	Apoptosis-inducing factor mitochondrial	3.75	7.73E−10
CPIJ007305	Conserved hypothetical protein	3.72	1.61E−10
CPIJ018624	Glutathione S-transferase 1	3.64	4.20E−25
CPIJ005645	Heat-shock protein 22	3.63	1.38E−08
CPIJ003813	Pickpocket (chemoreceptor)	3.57	1.39E−10
CPIJ008659	Metalloproteinase putative	3.53	4.00E−12
CPIJ002726	Lipase 3 precursor	3.52	8.39E−18
CPIJ014889	Pyruvate dehydrogenase	3.47	3.65E−09
CPIJ016451	Crotonobetainyl-CoA dehydrogenase	3.40	1.37E−101
CPIJ008888	p53 and DNA damage-regulated 1	3.37	1.15E−28
CPIJ004369	UDP-glucuronosyltransferase 1–7C	3.31	3.74E−12
CPIJ014496	Transient receptor potential cation channel protein	3.30	1.33E−15
CPIJ004637	Glutactin	3.22	6.40E−67
CPIJ013192	Hypothetical protein	3.20	9.53E−08
CPIJ015075/CPIJ011244	Heat-shock protein 83	3.16	4.56E−17
CPIJ018427	Nuclear pore complex Nup93-1	3.14	6.45E−07
CPIJ017588	Peroxidase	3.13	5.69E−08
CPIJ011585	Glutamate–cysteine ligase	3.12	5.48E−12
CPIJ019704	Probable cytochrome P450 6a17	3.12	8.99E−09
CPIJ019567	Vegetatible incompatibility protein HET-E-1	3.08	3.22E−07
CPIJ002042	Translocon-associated protein subunit delta	3.04	5.36E−07

**Table 3:** Top most upregulated genes in the *Culex quinquefasciatus* larvae from the RIAB59 resistant colony, revealed by the RNA-seq analysis

Gene_id	Description	Log2 fold change	*P*-value
CPIJ014172	2-hydroxyacyl-lyase 1	5.33	1.61E−24
CPIJ006306	Transient receptor potential channel	4.20	3.44E−14
CPIJ014195	Chitin binding protein	4.10	6.64E−11
CPIJ013708	Farnesoic acid 0-methyl transferase	3.83	2.49E−11
CPIJ002522	Farnesol dehydrogenase	3.76	4.07E−12
CPIJ004489	Sodium potassium calcium exchanger 5	3.75	1.95E−25
CPIJ014743	Guanine nucleotide-binding-like 3 homolog	3.71	1.81E−22
CPIJ019303	Transcription termination factor 2	3.45	2.06E−08
CPIJ015649	DNA-binding protein smubp-2 putative/Helicase mov-10-	3.38	4.23E−08
CPIJ011720	Venom dipeptidyl peptidase 4	3.18	3.49E−07
CPIJ010133	Structural maintenance of chromosomes protein	3.18	1.81E−08
CPIJ007000	Matrix metallo ase	3.15	4.68E−07
CPIJ018811	Apoptosis-inducing factor mitochondrial	3.15	4.96E−07
CPIJ013538	Ficolin-1	3.14	5.41E−07
CPIJ009033	Hexamerin/arylphorin subunit C223	3.13	8.45E−07
CPIJ010470	Transmembrane 19 isoform X1	3.11	7.19E−07
CPIJ000056	Hexamerin/larval serum 1 beta chain	3.05	3.16E−06
CPIJ004795	Mitochondrial carrier Rim2	3.04	7.47E−24
CPIJ019787	Angiopoietin-2/ficolin-3 precursor	3.03	1.63E−06

Among the upregulated mRNAs in the resistant colony (Table 3), the top most is the one encoding 2-hydroxyacyl-CoA lyase 1, an enzyme involved in lipid metabolism within the peroxisome [56, 57]. The identification among the top five upregulated transcripts of two genes encoding the enzymes farnesol dehydrogenase and farnesoic acid 0-methyl transferase, both implicated in the biosynthesis of the juvenile hormone in mosquitoes [58, 59], is another relevant finding in this profile. Upregulated DEGs found in the resistant colony also include genes associated with chromatin and DNA related processes (see CPIJ019303, CPIJ015649 and CPIJ010133). Other mRNAs listed in Table 3 include those encoding ion channels, a hypothetical protein with chitin binding domain and lesser known proteins such as the ficolins, a group of oligomeric lectins with roles in innate immunity in vertebrates [60, 61], and hexamerins, hexameric serum proteins whose levels have been shown to be altered upon infection in insects [62]. Transcripts encoding mitochondrial proteins and related to apoptosis are also found upregulated. Overall the differences between the most down- or upregulated genes are consistent with likely substantial changes in expression and abundance of individual proteins encoded by these genes in the resistant larvae.

### Categorization of differentially expressed transcripts

Considering the identification of several genes associated with lipid metabolism among the most downregulated transcripts in the RIAB59 resistant colony, a more detailed search for DEGs whose transcripts encoded related proteins was carried out. In addition to those genes listed in Table 2, several other transcripts encoding proteins associated with lipid catabolism were also seen to be downregulated with values of log2 fold changes between 1.9 (3.7-fold decrease) and 3 (8-fold decrease) (Additional file 1: Table S1). These downregulated transcripts include not only lipases, generally involved with the digestion of triacylglycerol molecules (CPIJ004230, CPIJ004226, CPIJ001036 and so on), but also enzymes which are predicted to be part of fatty acid and lipid degradation pathways, both in the mitochondria as well as in the peroxisome (CPIJ011600, CPIJ003870, CPIJ004138).

Next, to enable the identification of further metabolic pathways that can be associated with resistance to IAB59 and allow a wider understanding of the biological processes involved, the whole set of DEGs was assigned to defined KEGG metabolic pathways using a streamlined approach. Amongst the 1355 DEGs (Additional file 1: Table S1), 325 were then assigned to 123 KEGG pathways of which 36 showed terms specifically enriched for the downregulated DEGs from the resistant colony, of which four are involved in lipid metabolism (Table 4). These include pathways associated with the metabolism of sphingolipid (cqu00600), glycerophospholipid (cqu00564), ketone bodies (cqu00072) and other lipids (cqu00565). Two other pathways are linked with organelles associated with catabolism, phagosome (cqu04145) and peroxisome (cqu04146), with their downregulation being consistent with the previously described data. Several metabolic pathways are also indentified having multiple downregulated DEGs, including pathways involved with the metabolism of carbohydrates, nucleotides and amino acids. Regarding the upregulated genes from the resistant larvae, these were also assigned to 36 KEGG enriched pathways (Table 5). Signal transduction pathways, such as the mTOR (cqu04150) dependent pathway, and cellular processes such as autophagy (cqu04140) and the ubiquitin mediated proteolysis (cqu04120) are examples of biological processes that might be relevant for the resistant larvae due to their differential upregulation. It is also noteworthy that several pathways related to DNA replication and repair were enriched and mostly associated with upregulated transcripts, such as nucleotide excision repair (cqu03420), mismatch repair (cqu03430), homologous recombination (cqu03440) and non-homologous end-joining (cqu03450).

**Table 4:** KEGG pathways (36) with enriched downregulated terms in *Culex quinquefasciatus* larvae from the RIAB59 resistant colony

Kegg enriched pathway	Hits	*P*-value^a^	ID
Metabolism			
Global			
Microbial metabolism in diverse environments	9	1.14E−06	cqu01120
Carbon metabolism	4	8.01E−03	cqu01200
Carbohydrate			
Pyruvate metabolism	6	1.51E−06	cqu00620
Glycolysis/gluconeogenesis	4	4.76E−04	cqu00010
Citrate cycle (TCA cycle)	3	4.59E−03	cqu00020
Fructose and mannose metabolism	2	2.11E−02	cqu00051
Butanoate metabolism	2	1.91E−02	cqu00650
Glyoxylate and dicarboxylate metabolism	2	1.95E−02	cqu00630
Lipid			
Sphingolipid metabolism	3	4.06E−03	cqu00600
Glycerophospholipid metabolism	3	1.81E−02	cqu00564
Synthesis and degradation of ketone bodies	2	2.57E−03	cqu00072
Ether lipid metabolism	2	1.91E−02	cqu00565
Nucleotides			
Purine metabolism	13	1.26E−10	cqu00230
Pyrimidine metabolism	11	2.51E−10	cqu00240
Aminoacids			
Cysteine and methionine metabolism	3	2.58E−03	cqu00270
Arginine and proline metabolism	3	1.062E−02	cqu00330
Alanine, aspartate and glutamate metabolism	2	3.94E−02	cqu00250
Glycine, serine and threonine metabolism	2	4.85E−02	cqu00260
Other aminoacids			
Glutathione metabolism	5	1.16E−04	cqu00480
Selenocompound metabolism	2	5.56E−03	cqu00450
Xenobiotics biodegradation			
Metabolism xenobiotics by cytochrome P450	2	1.95E−02	cqu00980
Drug metabolism-cytochrome P450	2	1.95E−02	cqu00982
Genetic information processing			
Transcription			
Spliceosome	8	1.97E−05	cqu03040
RNA polymerase	4	3.40E−04	cqu03020
Translation			
Ribosome biogenesis in eukaryotes	12	4.82E−11	cqu03008
Ribosome	11	3.90E−08	cqu03010
RNA transport	10	6.57E−07	cqu03013
Folding/sorting/degradation			
Protein processing in endoplasmic reticulum	22	7.46E−24	cqu04141
RNA degradation	6	2.89E−05	cqu03018
Protein export	5	4.69E−06	cqu03060
Ubiquitin mediated proteolysis	4	1.18E−02	cqu04120
Environmental information processing			
Signal transduction			
FoxO signaling pathway	3	1.91E−02	cqu04068
Membrane transport			
ABC transporters	2	1.13E−02	cqu02010
Cellular processes			
Transport and catabolism			
Endocytosis	6	1.18E−04	cqu04144
Phagosome	5	2.11E−04	cqu04145
Peroxisome	4	4.59E−03	cqu04146

^a^*P-*values were corrected using the FDR (false discovery rate) approach, applied with the Benjamini-Hochberg procedure

**Table 5:** KEGG pathways (36) with enriched upregulated terms in *Culex quinquefasciatus* larvae from the RIAB59 resistant colony

Kegg enriched pathway	Hits	*P*-value^a^	ID
Metabolism			
Global			
Carbon metabolism	3	1.32E−02	cqu01200
Microbial metabolism diverse environments	3	3.25E−02	cqu01120
Biosynthesis of amino acids	2	4.75E−02	cqu01230
Carbohydrate			
Inositol phosphate metabolism	4	1.25E−04	cqu00562
Starch and sucrose metabolism	3	1.18E−03	cqu00500
Amino sugar & nucleotide sugar metabolism	3	5.95E−03	cqu00520
Fructose and mannose metabolism	2	9.98E−03	cqu00051
Lipid			
Glycerophospholipid metabolism	5	3.64E−05	cqu00564
Nucleotides			
Purine metabolism	3	4.19E−02	cqu00230
Other aminoacids			
Glutathione metabolism	3	4.46E−03	cqu00480
Co-factors/vitamins			
Folate biosynthesis	2	5.95E−03	cqu00790
Genetic information processing			
Transcription			
Spliceosome	5	1.30E−03	cqu03040
Basal transcription factors	2	2.97E−02	cqu03022
Translation			
RNA transport	5	2.16E−03	cqu03013
Ribosome biogenesis in eukaryotes	4	3.19E−03	cqu03008
mRNA surveillance pathway	3	1.27E−02	cqu03015
Folding/sorting/degradation			
RNA degradation	4	9.87E−04	cqu03018
Ubiquitin mediated proteolysis	8	1.69E−07	cqu04120
Protein processing in endoplasmic reticulum	4	6.61E−03	cqu04141
Replication and repair			
Base excision repair	5	3.33E−07	cqu03410
Fanconi anemia pathway	5	1.14E−05	cqu03460
Nucleotide excision repair	5	1.29E−05	cqu03420
Homologous recombination	4	6.85E−05	cqu03440
Mismatch repair	3	6.69E−04	cqu03430
DNA replication	3	2.61E−03	cqu03030
Non-homologous end-joining	2	2.17E−03	cqu03450
Environmental information processing			
Signal transduction			
FoxO signaling pathway	7	1.47E−07	cqu04068
mTOR signaling pathway	6	1.47E−07	cqu04150
Phosphatidylinositol signaling system	3	3.52E−03	cqu04070
Wnt signaling pathway	3	7.24E−03	cqu04310
Jak-STAT signaling pathway	2	7.60E−03	cqu04630
MAPK signaling pathway-fly	2	9.28E−03	cqu04013
TGF-beta signaling pathway	2	1.63E−02	cqu04350
Cellular processes			
Transport and catabolism			
Endocytosis	6	2.52E−05	cqu04144
Autophagy	2	7.60E−03	cqu04140
Organismal systems			
Development			
Dorso-ventral axis formation	2	1.27E−02	cqu04320

^a^*P-*values were corrected using the FDR (false discovery rate) approach, applied with the Benjamini-Hochberg procedure

**Fig. 2:**
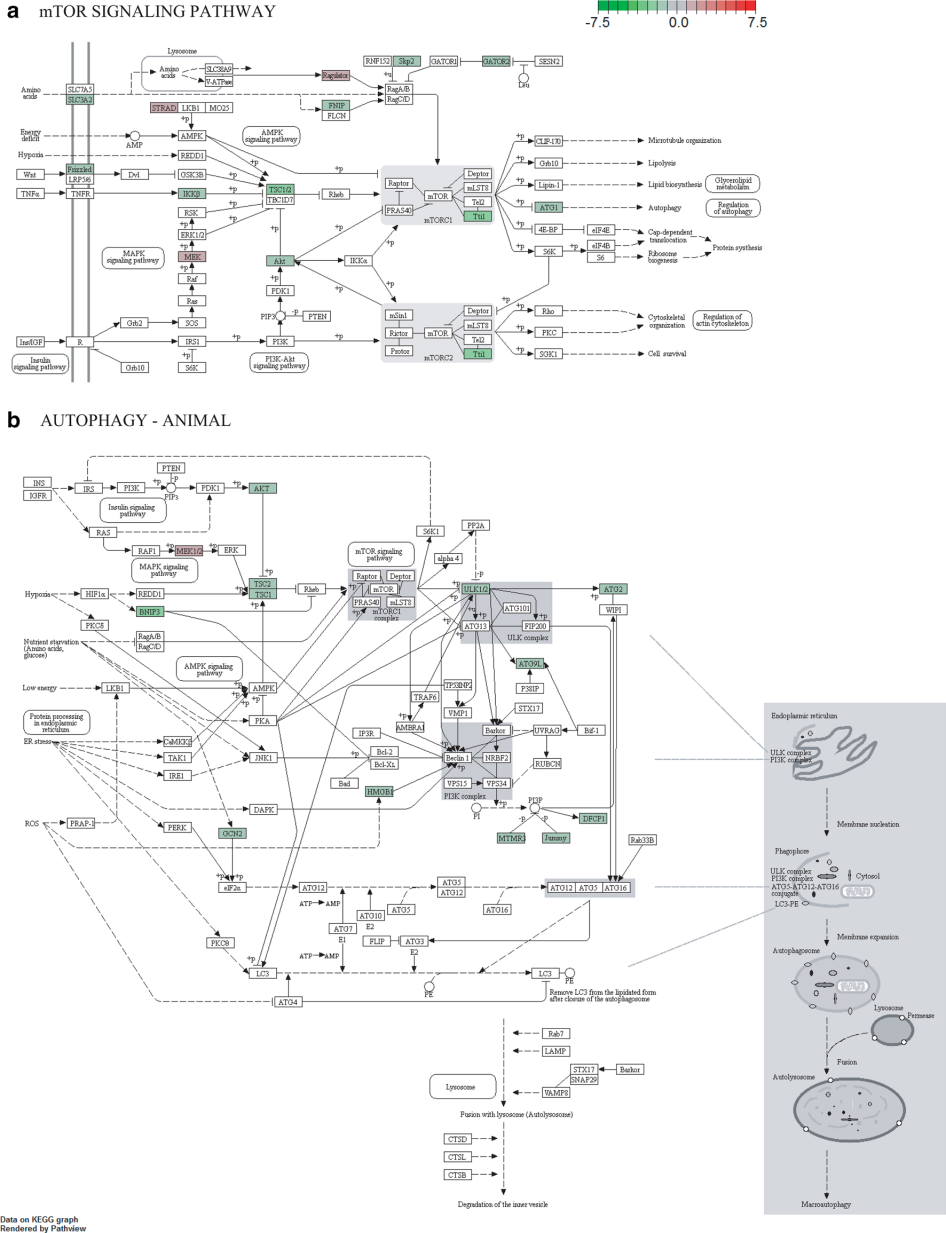
KEGG signaling pathways of mTOR (**a**) and autophagy (**b**) with differentially expressed genes in *Culex quinquefasciatus* larvae from a *Lysinibacillus sphaericus* resistant colony (RIAB59). Upregulated and downregulated genes are represented as green and red rectangles, respectively. Non-colored rectangles denote proteins which were not sequenced, not differentially expressed or do not have homologs annotated in the current *Cx. quinquefasciatus* genome

We also opted to examine selected KEGG pathways in order to have a clearer idea of the impact of the upregulated or downregulated DEGs. For this analysis the DEGs were individually assigned to high quality KEGG pathways annotated for *Cx. quinquefasciatus*, providing a more detailed analysis of the effects upon each pathway and including DEGs which might have been missed by the first approach. For some pathways, however, genes were detected coding for proteins that are involved either in the activation or inhibition of processes within the same pathway and the final outcomes on the regulation of those pathways are difficult to determine, although the data clearly indicate that these processes are under strong modulation. The mTOR pathway, for instance, showed eleven upregulated and three downregulated genes in the resistant colony (Fig. 2a). The autophagy pathway, in contrast, showed 12 upregulated and one downregulated gene with a more consistent upregulated profile (Fig. 2b). Other examples include pathways associated with DNA replication and repair, where several upregulated transcripts related to these pathways are found (Additional file 1: Table S1), although not included in Table 3 due to smaller values of log2 fold changes. These include transcripts encoding the DNA polymerase delta catalytic subunit (CPIJ018287), the DNA repair protein Rad50 (CPIJ004115) and the replication factor C/RFC (CPIJ017892), all found represented in two or more of the KEGG maps related to DNA metabolism (Additional file 3: Figure S1). Overall the KEGG analysis proved to be a usefull resource in order to produce a functional view of the differential expression profile of the resistant larvae, with analysis of the up- and downregulated genes being more informative. However, it is important to notice that relevant DEGs can be absent in these pathways. One example is the *cqm1* gene, of great functional relevance in this study but is missing from the currently available KEGG maps.

### STRING evaluation of protein-protein interactions among DEGs

**Fig. 3:**
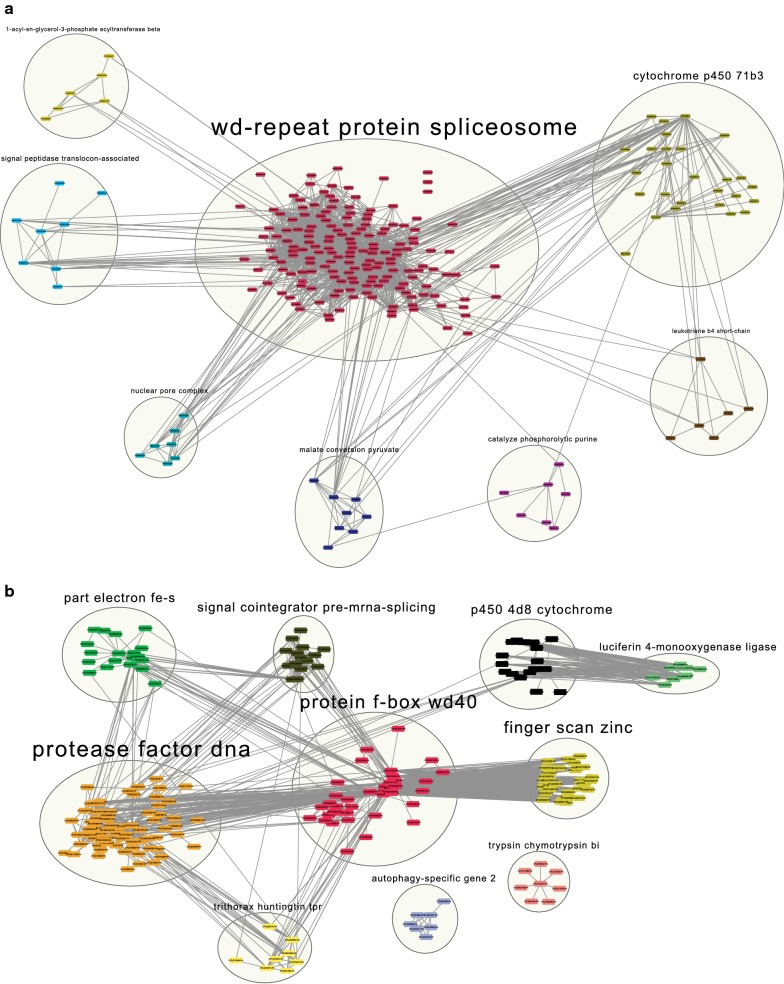
STRING clusters which display the functional protein-protein interaction of differentially expressed genes of *Culex quinquefasciatus* larvae from a *Lysinibacillus sphaericus* resistant colony (RIAB59). **a** Downregulated genes. **b** Upregulated genes

In order to analyze our findings at a different functional level, the DEGs were also analyzed with the STRING tool which provides a functional protein-protein interaction view of the data. The DEGs genes formed distinct clusters named according to their representation in the DEGs gene annotation (Additional file 4: Table S3). A set of downregulated genes (239) grouped in eight clusters (Fig. 3a), of which 169 formed a single cluster designed as “Wd-repeat protein spliceosome”, with a marked and concentrated network of molecular interactions. This major cluster is formed by genes encoding heat-shock proteins and ribonucleoproteins and displays interactions with all other clusters including the “cytochrome p450 71b36”, the second most represented one (28 genes), as well as six other smaller clusters (6–8 genes) which include the “catalyse phosphorolytic purine” were the vanin 1/pantetheinase gene is found. Considering a set of upregulated 226 genes in the resistant larvae, the analysis grouped them into ten clusters, some of which are in agreement with the pathways identified by the KEGG analysis (Fig. 3b). The “protease factor DNA” cluster includes the highest gene number of DEGs (69) and several of them are related to DNA metabolism, such as replication, repair, and chromosomes maintenance. Similarly to “DNA protease factor” the “protein f-box wd40” cluster group genes related with DNA metabolism and include some coding for proteins with cell cycle roles. Other clusters may also be related to specific metabolic processes enhanced in the resistant larvae, some of which were also implied by the analyses of KEGG pathways. Noteworthy are several of the genes found as part of the fourth cluster, “part electron fe-s”, which code for protein kinases responsible for cell signaling and protein regulation, implying changes in cell signaling and again the cell cycle as important for the resistance phenotype. Another interesting finding from the STRING data is the identification of clusters of completely distinct cytochrome p450 genes among both downregulated (cytochrome P450 71 b3) and upregulated (P450 4d8 cytochrome) DEGs. These may indicate marked changes in the expression of defense proteins by the resistant larvae.

### Validation of differential gene expression

**Fig. 4:**
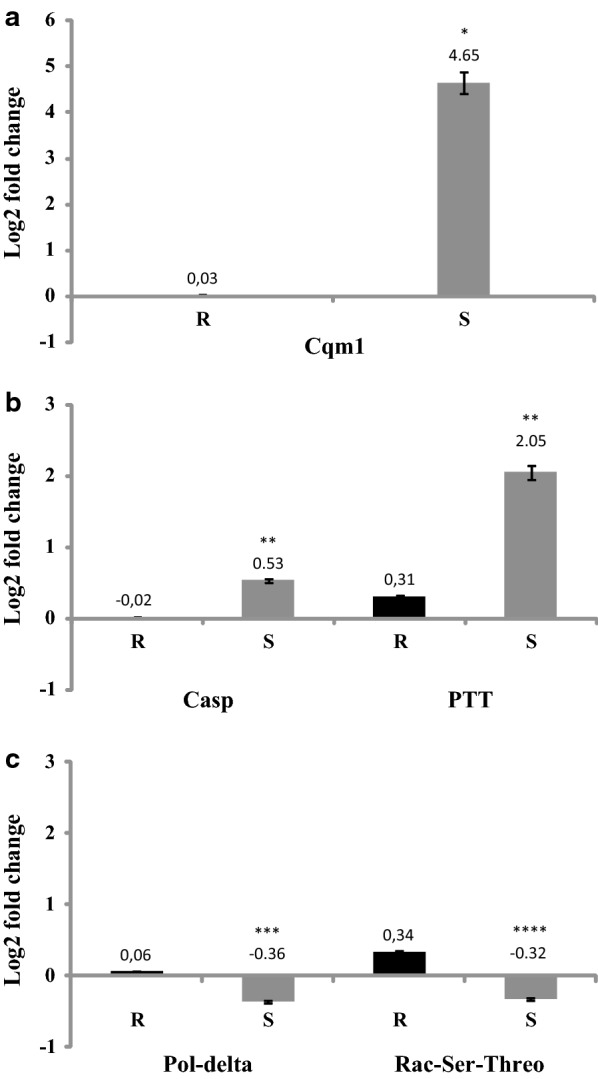
Relative expression levels of *Culex quinquefasciatus* genes from a *Lysinibacillus sphaericus* resistant larvae (R) compared to a susceptible ones (S), by quantitative real-time PCR. **a** Cqm1 gene. **b** Caspase-3 (Casp) and pantetheinase (PTT) genes. **c** DNA polymerase delta catalytic subunit (Pol-delta) and Rac serine/threonine kinase (Rac-Ser-Threo) genes. Gene expression levels are relative to those from the endogenous control gene *18S* used for normalization. Means and standard errors were obtained from three biological replicates. *P < 0.0001, **P < 0.008, ***P < 0.005, ****P < 0.05

To validate the RNA-seq data and the analyses derived from them, five DEGs were selected to have their relative expression profile evaluated by quantitative real time RT-PCR, comparing the resistant RIAB59 and the susceptible colonies. The selection considered both down- and upregulated DEGs in the resistant larvae, their potential biological relevance and also included genes with different ranges of log2 fold changes. The *cqm1* gene (CPIJ013173) was first chosen, since it is the internal marker for the resistant phenotype to the Bin toxin. Indeed, the evaluation of its relative expression levels by the qRT-PCR analysis showed a marked reduction in the resistant larvae (log2 fold change of 4.6 or a 25-fold decrease), as expected and confirming the RNA-seq results (Fig. 4a). The next two targets were chosen from the repertoire of downregulated genes, the vanin 1 pantetheinase (CPIJ017593) and caspase 3 (CPIJ012580). Pantetheinase showed the highest values of downregulation (log2 fold change of 7.1 or a 128-fold decrease) while the caspase 3 gene was selected due to the fact that it has an important role as a marker of apoptosis, despite the lower score of downregulation seen by us (log2 fold change of 2.1 or 4-fold decrease). Their relative transcription quantification performed by qRT-PCR confirmed reductions in levels of both mRNAs (log2 fold change of 2.0 or a 4.1-fold decrease for the pantetheinase and log2 fold change of 0.5 with a 1.4-fold decrease for the caspase transcript), with the reduction in levels of the pantetheinase mRNA being not as strong, for reasons unknown (Fig. 4b). For the last two genes, in addition to the scores of log2 fold changes, another relevant criteria for selection was their upregulation and participation in several KEGG pathways. The chosen Rac serine/threonine kinase gene (CPIJ007754) participates in five pathways including FoxO, mTOR and autophagy, while the DNA polymerase-delta gene (CPIJ018287) is a component of eight pathways related to DNA recombination/repair. For these genes the values of log2 fold changes according to the RNA-seq analysis for the resistant larvae (from Additional File 1: Table S1) were 1.35 (2.5-fold increase) and 2.4 (5.3-fold increase), respectively, with equivalent small but significant (*P* < 0.05) increases in expression, confirmed by the qRT-PCR (Fig. 4c). Overall these analyses then confirm that the differential expression patterns found for these selected genes with the qRT-PCR are in agreement with the respective profile revealed by RNA-seq.

## Discussion

Resistance of *Cx. quinquefasciatus* to the Bin toxin from *L. sphaericus* is one of a few examples where resistance can be directly linked to mutations in a gene coding for the toxin receptor, in this case the Cqm1 maltase [7, 25–29]. Indeed, the expression pattern seen here for the *cqm1* transcripts in resistant larvae, in agreement with the previous biological and molecular data [26, 36, 38], was a remarkable marker of Bin resistance in view of the substantial downregulation detected for this mRNA. The selection of resistance, however, possibly involves further changes and this study has identified several other genes and pathways whose expression are substantially altered in the resistant larvae. These are most likely associated with the strong resistance phenotype to Bin (RR > 3000-fold) rather than a response to the Cry48Aa/Cry49Aa toxin whose profile of low-resistance profile (RR~15-fold) detected in this study did not allow a reliable analysis. Nevertheless it is not possible to rule out that some differential responses exhibited by RIAB59 larvae could be related to their low-resistance to the Cry48Aa/Cry49Aa toxins. These changes could not be necessarily related to receptors, since Bin and Cry48Aa/Cry49Aa toxins do not depend on the same binding sites [34, 63], but eventually to other pathways specifically associated to their mode of action.

While the key role of Cqm1 as the *Cx. quinquefasciatus* receptor for the Bin toxin is known, candidate midgut ligands of the Cry48Aa/Cry49Aa toxin have only recently been identified, but have not yet been functionally validated as specific receptors. Those putative ligands include aminopeptidases/metalloproteases, alkaline phosphatases and maltases, some of them orthologs to molecules previously identified as receptors for other insecticidal Cry toxins [35]. In this study, however, none of these candidates showed any significant changes in expression at the transcriptional level. This might be linked to the low resistance level to the Cry48Aa/Cry49Aa toxin detected here for the RIAB59 larvae, as described above, although mutations changing binding sites would not be detected by our approach nor would changes in genes inducible by exposure to the toxins. A possible exception, nevertheless, was the vanin 1/pantetheinase, since this protein was found among the previously identified candidate and the downregulation of its gene detected here is higher than that observed for the *cqm1* gene. At this stage, however, pantetheinase’s downregulation might rather reflect changes in the lipid metabolism associated to the resistance to the Bin toxin. This is due to the fact that vitamin B5, the product of the vanin 1/pantetheinase, is an important cofactor for lipid biogenesis and degradation [48]. In addition, an RNA-seq study using larvae resistant to the 2362 strain that produces Bin toxin only showed a similar decrease in levels of pantetheinase mRNAs (our unpublished data), which strongly suggests its association with the Bin toxin resistance. Another protein with possible alternative roles in the resistance phenotype is apolipoprotein D (ApoD), a carrier of lipophilic molecules that has been shown to play a critical role in anti-oxidation and anti-apoptosis [64]. ApoD is a member of the lipocalin protein family, consisting of a large and diverse group of extracellular proteins with several functions in cellular regulation and homeostasis [65]. ApoD has also recently been shown to have important functions associated with stress response, longevity regulation and control of metabolism [64].

The downregulation of genes related to lipid catabolism in the resistant colony corroborates a previous finding which detected a remarkable accumulation of lipid inclusions in the midgut epithelial cells from non-treated fourth instar larvae from another *Cx. quinquefasciatus* colony (R2362) resistant to the Bin toxin [15]. Lipid synthesis in mosquitos has been observed as a response to bacterial pore-forming toxins [66, 67] and recently the accumulation of lipid droplets observed in *Ae. aegypti* challenged with viruses and bacteria was also clearly correlated with its involvement in mosquito immunity [68]. An intriguing observation is the strong upregulation of the gene encoding peroxisomal 2-hydroxyacyl-CoA lyase, presumably also involved in lipid degradation. In animal cells, fatty acid oxidation can occur in both mitochondria and peroxisomes [69] with these two ubiquitous organelles being metabolically connected, so that any alteration in mitochondrial function may induce changes in peroxisomal physiology [69–71]. It would then not be surprising if a chronic mitochondrial dysfunction induced as an adaptive host response to the action of pore-forming toxins might lead to a compensatory change in the peroxisomes as a consequence of the resistance event.

Upregulation of genes involved in DNA metabolism was one of the most marked differential expression feature exhibited by the resistant colony, and indicates that the resistant colony developed an expression profile to respond to DNA damage. The mode of action of bacterial pore forming toxins also involves apoptosis and direct evidence has been specifically provided for Bin [17] and Cry toxins [72]. The intrinsic apoptotic pathway is specifically activated in response to mitochondria damage, resulting in the cytochrome C release from these organelles and caspases activation that leads to other events, including DNA degradation [72]. A higher expression of caspase 3 in susceptible larvae treated with Bin toxin was an effect caused by this toxin to the cells [73]. The action of Bin toxin induces autophagy in the midgut cells and subsequent cytoplasmic vacuolization as it has been well documented in larvae subjected to Bin treatment [16, 17]. In resistant larvae several genes from the autophagy pathway (cqu04140) show differential regulation and these may be related to the lower capacity by the Bin toxin to act upon the resistant cells and provoke autophagy.

The identification of several genes and pathways whose expression are substantially changed in the resistant colony provides a framework for understanding some of the mechanisms involved in the resistance to *L. sphaericus* toxins, mainly Bin, as well as more generall mechanisms required by the larvae to survive the exposure to the bacterium. Indeed, a broader profile of features associated with resistance of such larvae, other than the major gene already shown to be required for resistance to the Bin toxin, is essential for the understanding the strategies used by these insects for their survival and for their ability to overcome the toxic effects. This aspect is of particular importance for the management of insecticide resistance in the field.

## Conclusions

The dataset provided here shows that the resistant RIAB59 larvae is associated with a gene expression profile distinct from susceptible larvae. This is correlated not only with lack of the receptor which primarily confers the Bin resistance status, but also with metabolic pathways that might enhance the survival of the larvae to the effects mainly caused by the Bin toxin from the IAB59 strain. DEGs in pathways associated with lipid catabolism, DNA metabolism, apoptosis and immune response were specifically regulated and are likely to be associated with the response to *L. sphaericus* and with the stable resistance phenotype. This study also highlights other adaptative features that are likely to be crucial for the capacity of these individuals to successfully maintain this phenotype on a long-term basis.

## Additional files

**Additional file 1: Table S1.** Differentially expressed genes of *Culex quinquefasciatus* larvae from a *Lysinibacillus sphaericus* resistant colony (RIAB59) compared to a susceptible colony.

**Additional file 2: Table S2.** Primers used to perform qRT-PCR reactions to evaluate the expression of *Culex quinquefasciatus* genes.

**Additional file 3: Figure S1.** Examples of KEGG pathways that displayed mostly upregulated genes related to DNA synthesis and maintenance in *Culex quinquefasciatus* larvae from a *Lysinibacillus sphaericus* resistant colony compared to a susceptible one. a Nucleotide excision repair (cqu03420). b Mismatch repair (cqu03430). c Homologous recombination (cqu03440). d Non-homologous end-joining (cqu03450).

**Additional file 4: Table S3.** Description of STRING clusters found for downregulated and upregulated genes of *Culex quinquefasciatus* larvae from a *Lysinibacillus sphaericus* resistant colony (RIAB59) compared to a susceptible colony.

## Data Availability

The transcriptome dataset is available (release date 2019-09-30) at https://www.ncbi.nlm.nih.gov/sra/PRJNA551311.

## Abbreviations

Bin: Binary
Cqm1: *Culex quinquefasciatus* maltase 1
DEG: differentially expressed genes
GPI: glycosylphosphatidylinositol
KEGG: Kyoto Encyclopedia of Genes and Genomes
RIAB59: *Culex quinquefasciatus* strain resistant to *Lysinibacillus sphaericus* strain IAB59
RR: ratio of resistance
