# Supplementary material for: A differential transcriptional profile by Culex quinquefasciatus larvae resistant to Lysinibacillus sphaericus IAB59 highlights genes and pathways associated with the resistance phenotype
Source: Parasit Vectors. 2019 Aug 20;12:407. doi: 10.1186/s13071-019-3661-y (PMC6702717; doi:10.1186/s13071-019-3661-y)
Supplement: Supplementary file 2 — Additional file 2: Table S2. Primers used to perform qRT-PCR reactions to evaluate the expression of Culex quinquefasciatus genes. [file 13071_2019_3661_MOESM2_ESM.doc]

**Additional file 2: Table S2.** Primers used to perform qRT-PCR reactions to evaluate the expression of *Culex quinquefasciatus* genes.

| **Gene** | **Primers (5’-3’)^1^** | **Amplicon (bp)** |
| --- | --- | --- |
| 18S^2^ | F CGCGGTAATTCCAGCTCCACTA | 159 |
|  | R GCATCAAGCGCCACCATATAGG |  |
| CPIJ007754 (Rac-Ser-Threo) | F CACGGCTGCGGCTTCAGTG | 180 |
|  | R GCGTCCGGACGGTTCTTGTAC |
| CPIJ018287 (DNA Pol-delta) | F GAGAATCCTCCTCGGCCAGC | 191 |
|  | R CTTCGGACGGCTCCACTTG |
| CPIJ013173 (Cqm1) | F GAACCGGACTCGAAGGACTG | 228 |
|  | R CTCCGGCTGAATATCGTAGAAATC |  |
| CPIJ017593 (Pantetheinase) | F TCTTTGCCTGCACCGGGACCA | 228 |
|  | R CGAATGGTTTGCCATCCTTCGT |  |
| CPIJ012580 (Caspase 3) | F GGCACCAAGTCCACGTTCTC | 214 |
|  | R GAGTGATTGCTGTTGGTGAGTTGC |  |

^1^ Primers forward (F) and reverse (R) were designed using PrimerSelect (DNASTAR) from the sequences obtained in the VectorBase platform (www.vectorbase.org).

^2^  Endogenous control gene, sequence from Liu et al. (2011).
