# Supplementary material for: A differential transcriptional profile by Culex quinquefasciatus larvae resistant to Lysinibacillus sphaericus IAB59 highlights genes and pathways associated with the resistance phenotype
Source: Parasit Vectors. 2019 Aug 20;12:407. doi: 10.1186/s13071-019-3661-y (PMC6702717; doi:10.1186/s13071-019-3661-y)
Supplement: Supplementary file 3 — Additional file 3: Figure S1. Examples of KEGG pathways that displayed mostly upregulated genes related to DNA synthesis and maintenance in Culex quinquefasciatus larvae from a Lysinibacillus sphaericus resistant colony compared to a susceptible one. a Nucleotide excision repair (cqu03420). b Mismatch repair (cqu03430). c Homologous recombination (cqu03440). d Non-homologous end-joining (cqu03450). [file 13071_2019_3661_MOESM3_ESM.doc]

**Additional file 3: Figure S1**. Examples of KEGG pathways that displayed mostly up regulated genes related to DNA synthesis and maintenance in *Culex quinquefasciatus* larvae from a *Lysinibacillus sphaericus* resistant colony compared to a susceptible one. a. Nucleotide excision repair (cqu03420). b. Mismatch repair (cqu03430). c. Homologous recombination (cqu03440). d. Non-homologous end-joining (cqu03450).
